# Ague Cake

**Published:** 1861-08

**Authors:** 


### CHICAGO MEDICAL DISPENSARY.

Service, Prof. Wm. H. BYFORD, Attending Physician.

Reported for the EXAMINER.

I. *Ague-Cake.*—Patrick Gougens, æt. 16 years; has been under treatment some six months, and was presented at the clinic of July 13th, as a good illustration of that form of disease accompanying the intermittent, and, probably, some other forms of fever,—namely: the *enlarged spleen.*

*The patient's history* is briefly, as follows:—When about ten years of age, while residing at Ft. Hamilton, N. Y., he had an attack of fever and ague (intermittent fever,) which lasted about two years, and towards the close of which the body began to swell, and a lump was discovered in the left side. For this, little was done, until about twelve months since, when, at the suggestion of an old woman, he commenced taking the following decoction: a handful each of horse-radish, parsley-root and lignum-vitæ shavings, with a pint of white mustard seed, were boiled twenty-four hours in a half-gallon of hard cider; of this, half a glassful was taken three times a day. The swelling (ascites) subsided, and, with the exception of the "hard lump" in the left side, the boy has been pretty well since.

*An examination* by the class, revealed the following condition:—abdomen very much enlarged, particularly in the hypochondriac and epigastric regions; and on a more minute survey the boundaries of an enormously enlarged spleen were very clearly defined,—extending from under the false ribs to the crest of the ilium on the left side, and beyond the linea alba in the median line—completely filling up the whole of the hypochondriac and epigastric regions of the left side, and rendering the abdomen tense and hard as a board to the touch. The liver was also found to be considerably enlarged,—extending some three inches below the floating ribs on the right side, with a thin but hard edge, which dipped beneath the spleen in the epigastric region. The surface of the spleen is flat and even, not nodulated or irregularly elevated; the lower boundary may be felt, as a thin, hard edge, by pressing the abdominal walls forcibly towards the spine, and its right margin may be made to glide over the liver, a little to the right of the median line.

In commenting upon the case, the lecturer remarked that it seemed to be almost a necessary part of the pathology of the intermittent fevers, that the spleen should become involved, and so uniformly was this the case as to have led theorists and speculators to urge that these diseases commence in the spleen. This, however, is by no means clearly proven; nor is the pathological explanation entirely satisfactory,—that splenic disorders depend on a congested condition of the portal circulation, owing to the blood being driven in from the capillaries during the cold stage; the continuance of which congestion causes the hypertrophy,—a condition of affairs also affecting the liver with like results. The fact, however, that the cure of the fever does not cure these accompaniments; and the still stronger facts, that ague-cake is not unfrequently met with in persons who have resided in malarious regions where the intermittents prevail, and yet, who have never had an attack of the disease, shows the necessity for still further research. And this, following the clue given by this last class of cases, leads to the suspicion that the peculiar influence,—miasm, malaria or whatever it is,—which produces the intermittents, is also the cause of the enlarged spleen and liver. And this we accept as a satisfactory explanation. The effect produced on the blood, by chills and fever, is very apparent in the destruction of the red globules; whether proximately, in the mass of the general circulation, or secondarily, through failure in the functions of the spleen,—this last, caused partially by congestion and stasis of the portal circulation, but mainly by the morbific effect of the miasmatic poison,—is rendered doubtful, by our ignorance of the office of this organ.

The congestion, however, produces effects about which we can entertain no doubts, in damming up the blood in the large veins of the abdomen, soon followed by deterioration of the whole mass of that fluid, and the destruction of the equilibrium between its solid and liquid constituents,—the preponderance of the latter, tending towards extravasation and effusions, soon apparent in the more dependent parts of the body. This is the received explanation of the production of dropsies in chills and fever, and, doubtless, was the case with the boy before us. But dropsy may be produced in another manner,—by direct pressure on the trunks of large veins, either by enlarged organs near which they pass,—as in the anasarca of the lower extremities of pregnant women, produced by the pressure of the gravid uterus on the iliac veins,—or by aneurisms, hernias, etc. Any enlargement of the abdominal viscera is apt to produce such results; pressure on the inferior vena cava, causing œdema of the lower extremities, and on the mesenteric veins causing abdominal effusions or ascites. This latter, coupled with the existence of these large veins on the surface of the abdomen enables us to diagnose, pretty accurately, the point of pressure. We see these veins, arising in the thorax, spreading, in a tortuous manner, over the abdomen, and penetrating it in the iliac region, performing the duties of the mesenteric, in gathering the blood from the intestines. Pressure on the emulgent veins also produces complications, the most frequent of which is albuminuria, many cases of which I have clearly traced to this cause.

*In diagnosticating* enlarged spleen, care should be taken to make a thorough examination; strip the patient and examine thoroughly by palpation and percussion; and then, in connection with the history of the case, but little difficulty will be found in determining the character of the tumor. The history from the patient is valuable and important in this respect. Ascertain whether he has ever lived in a malarious region, and if so, how long; whether he had had intermittent, remittent or typhoid fever, previous to the abdominal swelling. Enlargements of the spleen or liver always commence to show the swelling in one of the hypochondriac regions, and this gradually extends downwards into the abdomen, and the patient, if intelligent, will so describe its progress. Inquiries as to the previous state of health, are also important, —as these disorders never occur while in absolute good health, though there may, it is true, be no serious illness. These points will enable you to avoid the error of one of our country practitioners, who recently sent me a case of ovarian tumor, the history of which, before making any examination, clearly pointed out the existence of a simple, harmless, enlarged spleen.

*The treatment* may be inferred from this last remark, to be one of time and hygienic observances, rather than actively medicinal. The patient's health must be kept at the highest possible point; his diet must be generous, but not stimulating, good beef and mutton, forming a large proportion of it; he must exercise freely, in the open air if possible, and yet must guard against exposure. Above all he must be removed at once from the influence of the miasmatic poison which, causing the trouble originally, is capable of maintaining its evil effects, and of neutralizing all attempts at cure. If medical interference be deemed necessary, you will fail if you resort to iodine or mercurials; but the agent, so specific in the intermittents,—*quinine*,—will be found most beneficial. This may be given in grain doses, three times a day, for two or three weeks, then intermit for a like space, and renew; this seems to be a necessary precaution against the action of the salt wearing out by long use.

This lad's occupation (that of a news-boy) keeps him in the open air a good deal, and the observance of the hints already given, are gradually and steadily improving his condition, so that now, aside from the somewhat aldermanic proportions of his abdomen, he is comfortable, and enjoys a very good degree of health.

II. *Triple Hernia.**—I show you here an interesting case presenting the unusual combination of three congenital hernias, of which two are scrotal, and one umbilical. Congenital hernias are the result of arrested development, in consequence of which the inguinal or umbilical canals remain open after birth, instead of closing at the proper period. The scrotal hernias, you see, are quite large, but easily reduced, and in nothing different from other tumors of the same kind. The umbilical rupture is small and soft. It is common to meet this congenital defect in any one of the three places which you see here occupied, but it is very rare to find the patient having them all at once.

Fortunately, although the canals in question occasionally remain open until after birth, yet the fœtal tendency to close them still continues for many months. Hence, in these cases we may almost always obtain a radical cure without any operative interference. All that is necessary is to keep the hernia constantly reduced, so that the presence of the intestine shall not prevent the contraction of the canal. By this simple

* Service, Prof. E. ANDREWS, Attending Surgeon.

